# To what extent is multi-morbidity associated with new onset depression in patients attending cardiac rehabilitation?

**DOI:** 10.1186/s12872-019-1245-6

**Published:** 2019-11-14

**Authors:** Serdar Sever, Patrick Doherty, Alexander Stephen Harrison, Su Golder

**Affiliations:** 1 Department of Health Sciences, Faculty of Science, University of York, York, UK; 2 Department of Health Sciences, Faculty of Science, University of York, ATB/255 Seebohm Rowntree Building, York, UK

**Keywords:** Cardiovascular disease, Cardiac rehabilitation, Depression, New onset depressive symptoms, Comorbidities, Observational study

## Abstract

**Background:**

Depression is associated with increased mortality and poor prognosis in patients with cardiovascular disease (CVD). However, little is known about the patient characteristics associated with new onset post heart event depressive symptoms, specifically medical comorbidities, among cardiac rehabilitation (CR) participants. Therefore, this paper examines the comorbidity profile and characteristics associated with new onset depressive symptoms in patients attending CR.

**Methods:**

An observational study using the routine practice data of British Heart Foundation National Audit of Cardiac Rehabilitation (NACR) from the last six years between April 2012 and March 2018. Patients with new onset post heart event depression and no previous documented history of depression were selected as the study population. An independent samples t-test and chi square tests were used to compare the association between new onset depressive symptoms and patient variables including demographics, clinical measures and comorbidities. A binary logistic regression was conducted to investigate the predictors of new onset depressive symptoms employing log-likelihood ratio statistic.

**Results:**

The analyses included 109,055 CR patients with new onset depression measured by Hospital Anxiety and Depression Scale (HADS). At baseline assessment, comorbidity measures associated with new onset depressive symptoms were increased total number of comorbidities and a range of comorbidities - including diabetes, angina, arthritis, chronic back problems, asthma, stroke, anxiety, rheumatism, claudication, osteoporosis, chronic bronchitis and emphysema. After multivariate adjustments were done, at the start of CR, the significant predictors of new onset depressive symptoms were physical inactivity, high HADS anxiety score measurement, increased weight, total number of comorbidities, diabetes, stroke, chronic back problems, being from areas with higher levels of social deprivation, being single, and male.

**Conclusion:**

The research findings establish new insights into the association between patient demographic and clinical variables across a range of comorbidities in patients with new onset post heart event depressive symptoms. At the start of CR, patients with new onset depressive symptoms need to be assessed skilfully as they tend to have a complex multi-morbid presentation linked to psychosocial risk factors known to hinder CR engagement.

## Background

Depression is common after cardiovascular disease (CVD) and is associated with worse prognosis and increased mortality rates [1]. Nearly 20% of patients experience major depression after an acute heart event [2]. The co-occurrence of cardiovascular disease and depression is of concern to health care providers and commissioners considering its association with increased health care utilisation costs and hospital readmission rates [3]. Depression is also associated with treatment noncompliance, loss of productivity, and disability in patients with CVD [4].

Cardiac rehabilitation (CR) is a multicomponent programme which aims for secondary prevention as well as improving patients’ psychosocial health [5]. CR is effective and recommended for CVD patients according to a recent Cochrane review [6]. In addition, some meta-analyses have shown that CR reduces depressive symptoms [7, 8]. Recent CR and prevention guidelines recommend the assessment of depression under the virtue of being a multicomponent intervention and as a risk factor for poor medical outcomes in CVD patients [1, 5, 9, 10]. In pursuance of the management of psychosocial health, in the UK, the Hospital Anxiety and Depression Scale (HADS) is used at baseline CR to make adjustments to the intervention based on patient requirements and individual goals [11]. Furthermore, HADS is a reliable and validated tool for the assessment of depressive symptoms in CVD patients in different countries including the UK [12, 13].

Recent studies are particularly focused on the time onset of depressive symptoms in relation to adverse cardiac events and mortality outcomes [14–17]. Some studies have shown that patients with history of depression prior to a heart event had increased cardiac morbidity and risk for mortality [14, 15], however, other research has shown that new onset depressive symptoms after a heart event was particularly related to increased mortality and adverse cardiac events [16, 17]. A recent paper using NACR data further developed this literature and found that patients with prior history of depression and who present with high level of depressive symptoms at the start of CR tend to have increased weight, BMI, anxiety symptoms, smoking, physical inactivity, and they were younger and more likely to be single compared to patients who have depression history but absence of depressive symptoms at the start of CR [18]. This study aims to build on the previous work done in this field with a unique population yet to be addressed, patients without history of depression, meaning patients with new onset post heart event depressive symptoms, to examine which patient characteristics are associated with developing new onset depressive symptoms in relation to cardiovascular disease. Therefore, this study examines the demographic and clinical characteristics associated with new onset depressive symptoms in patients attending CR. In addition, prior studies were unable to include different types of comorbidities and examine their association with depressive symptoms in CR patients which have been included in this study. Thus, this study is the first to further investigate the comorbidity profile of patients to enable CR practitioners to better understand the patient profile and association of a variety of comorbidities at baseline, prior to the start of CR, with new onset depressive symptoms in the UK CR context.

## Methods

This study used a robust retrospective observational methodology to examine the association of medical comorbidities and individual patient characteristics with new onset depressive symptoms in CVD patients attending a CR assessment. This study has been reported applying the strengthening the reporting of observational studies in epidemiology (STROBE) checklist [19].

### Data collection

The analysis was conducted using patient data in the NACR database. The NACR aims to monitor CR services and improve the quality of UK CR programmes. In order to realize this aim individual patient level data are collected by CR programmes under section 251 approval of the NHS Act 2006 and entered into a secure online system hosted by NHS digital. NHS digital has approval to collect identifiable patient data which are then anonymised before data can be extracted and made available to the NACR for audit purposes. Therefore, there was no need to gain patient consent from individuals due to this data governance process. Data governance agreement between NHS Digital and NACR is reviewed every year by NHS digital. In the current study as the data is used in line with the NACR purposes and complies with data protection regulations, no separate NHS ethical approval was required. There are 229 CR services entering the data electronically which is 80% of all programmes [11]. The data includes patients’ initiating event, demographics, risk factors, treatment, medication and outcomes who undergo CR in the UK.

### Participants

The NACR data between the dates – 1 April 2012 to 31 March 2018 – were extracted and analysed. The study population included patients with myocardial infarction (MI) and heart failure (HF) and those who receive treatment of percutaneous coronary intervention (PCI) and coronary artery bypass graft (CABG) as recommended in the clinical guidelines [20, 21]. From the overall population of NACR cohort (*N* = 277,521), 152,068 patients (55%) had their HADS measurements recorded. All the eligible patients (*N* = 109,055) who had baseline HADS assessments recorded in CR, and who did not present with prior history of depression, were selected as participants during the study period.

### Measures

Patients who had no reported prior history of depression and possessed a HADS measurement at the start of CR were screened through the NACR data set. This approach defined the eligible population that constituted our study sample.

#### Hospital anxiety and depression scale

The HADS is an assessment tool consisting of self-answered questions which is used for the measurement of depressive symptoms. Psychosocial health measurements, including HADS, are recommended to be assessed pre and post CR to adjust CR services to the participants [5]. There are overall 14 items in HADS, 7 of the items cover anxiety symptoms and the remaining 7 covers depression. Every item is scored between 0 and 3, meaning separate anxiety and depression scales can be scored between 0 and 21. HADS is a reliable and valid measure for the assessment of depression and anxiety symptoms not only in general population but also in CVD patients [12, 13, 22]. HADS depression measurement, assessed at the start of CR, employed for the analysis and the clinical cut off point of 8 was used to categorize patients into absence of new onset depressive symptoms (< 8) and presence of new onset depressive symptoms (≥ 8) groups [13]. Afterwards, participants with HADS < 8 and HADS ≥8 were compared with the analysis in patients with no history of depression. HADS anxiety scores of the patients with absence and presence of new onset depressive symptoms were also compared. The HADS and other measurements were explained in detail elsewhere [18].

#### Total number of comorbidities and comorbidity types

Total number of comorbidities variable is calculated as the sum of the number of comorbidities present in a patient. Comorbidities defined as the medical history of conditions in the NACR data which is confirmed by CR providers. The comorbidities included in the current study are hypertension, hypercholesterolemia, diabetes, angina, arthritis, osteoporosis, asthma, chronic bronchitis, emphysema (COPD), cancer, rheumatism, stroke, claudication, chronic back problems, anxiety, family history of heart disease and erectile dysfunction.

#### Other variables

Age, gender, marital status (partnered/single) were the patient demographics used in the analysis. In addition, another patient demographic was The English Index of Multiple Deprivation (IMD) which is a measure of deprivation applied in England. Seven domains are used to construct IMD measure: employment, health deprivation and disability, income, crime, barriers to housing and services, living environment, education skills and training [23]. There are 32,844 sub-areas ranked from the most to least deprived areas. In our analyses, IMD was used to categorise patients into two quintiles where first quintile, reported as ‘lowest quintile’, represents the most deprived areas and other quintiles categorised as the less deprived. Smoking variable was categorised to current smoker or non-smoker. Other variables were, weight (kg), body mass index (BMI), moderate physical activity (150 min a week), and vigorous physical activity (75 min a week). Preliminary baseline assessments and the literature search were decisive for the inclusion of these variables. These variables have been explained in detail in a previous publication [18].

### Statistical analysis

The data was analysed using the IBM statistical package for social sciences (SPSS) software statistics version 25 (New York, USA). Patients without prior history of depression who have new onset pre HADS assessments constituted the study population. A *p* value of ≤0.05 was considered to be statistically significant. Means, standard deviations, and percentages were used to present summary statistics. T-tests were employed to investigate the mean difference for presence of new onset depressive symptoms and absence of new onset depressive symptoms grouped by age, total number of comorbidities, weight, BMI, and HADS anxiety. Chi-square tests were used to investigate the association between new onset depressive symptoms and smoking, gender, IMD, physical activity, marital status, and variety of comorbidities. Effect sizes were reported as Cohen’s d for continuous variables and phi for categorical variables. A binary logistic regression was conducted to examine the statistically significant predictors of new onset depressive symptoms (HADS ≥8) at the start of CR in multivariate analysis.

## Results

**Fig. 1:**
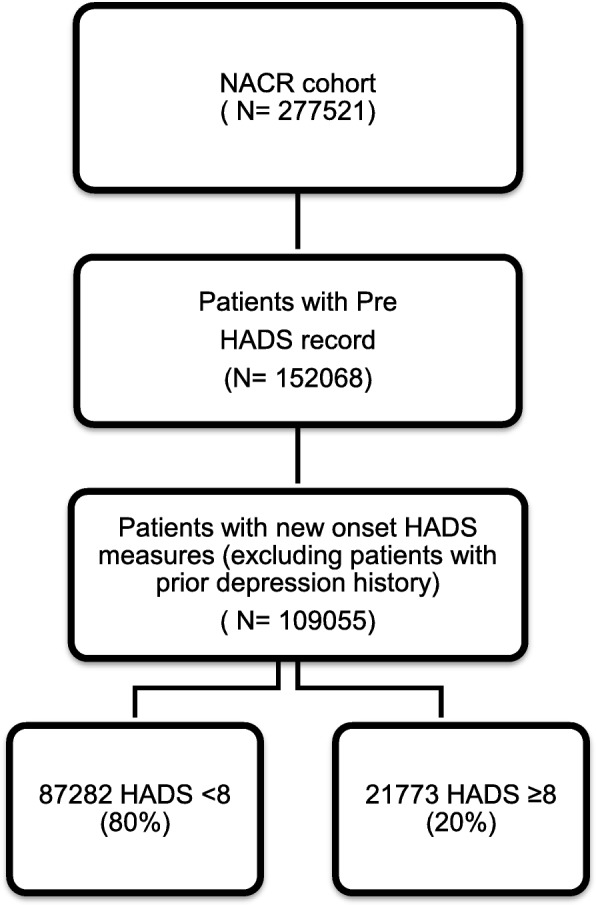
A flow diagram for study sampling

A total of 109,055 patients without prior history of depression who had started CR with valid pre HADS assessments constituted the study population. Of these 109,055 participants 20% were present with new onset depressive symptoms (HADS≥8) and 80% had absence of new onset depressive symptoms (HADS< 8). The total population during the study time period and the study sample size has been shown in the flow diagram in Fig. 1. Baseline characteristics of patients according to their HADS levels (HADS≥8 presence of new onset depressive symptoms, HADS< 8 absence of new onset depressive symptoms) are presented in Table 1.

**Table 1:** Baseline characteristics with t-tests for mean difference for presence and absence of new onset HADS depressive symptoms groups

Variables	HADS < 8 group (*n* = 87,282)	HADS ≥ 8 group (*n* = 21,773)	Difference	95% CI	*P*	d
	Mean ± SD	Mean ± SD				
Age	66.24 ± 10.96	64.01 ± 11.69	−2.23	− 2.40 to − 2.06	< 0.001	0.20
Total Comorbidities	2.36 ± 1.46	2.60 ± 1.58	0.24	0.22 to 0.27	< 0.001	0.16
Weight	82.67 ± 16.86	83.80 ± 19.05	1.13	0.84 to 1.42	< 0.001	0.07
BMI	28.21 ± 4.97	29.00 ± 5.77	0.79	0.70 to 0.88	< 0.001	0.16
HADS Anxiety Score Measurement	4.48 ± 3.37	9.86 ± 4.12	5.38	5.33 to 5.44	< 0.001	1.52

*SD* standard deviation, *CI* confidence interval, *d* Cohen’s d

Participants with new onset depressive symptoms group had a higher total number of comorbidities, increased anxiety scores, higher BMI, increased weight, and were younger compared to those with an absence of new onset depressive symptoms group.

In addition, patients with new onset depressive symptoms at the start of CR were more likely to be female, physically inactive, more likely to smoke, be from areas with higher social deprivation and single. Chi-square test results are shown in Table 2.

**Table 2:** Results from chi square test of association for presence and absence of new onset HADS depressive symptoms groups

Variables	HADS < 8 Group % (*n* = 87,282)	HADS ≥ 8 Group % (*n* = 21,773)	*P*	Effect size
Female	24.9	30.6	< 0.001	0.05
150 min. Physical Activity a Week (Yes)	44.0	26.4	< 0.001	0.14
75 min Vigorous Exercise a Week (Yes)	9.3	4.7	< 0.001	0.07
Smoking (Yes)	6.6	11.8	< 0.001	0.08
Partnered	78.2	72.7	< 0.001	0.05
IMD (Most deprived)	11.4	18.0	< 0.001	0.08

Once the comorbidity profile of the patients has been investigated, at baseline assessment, patients with presence of new onset depressive symptoms were more likely to have the medical comorbidities of angina, arthritis, diabetes, rheumatism, stroke, osteoporosis, chronic bronchitis, emphysema, asthma, claudication, chronic back problems, and anxiety compared to patients with absence of new onset depressive symptoms. However, patients with new onset depressive symptoms were less likely to have cancer, family history of CVD and hypercholesterolemia. The comorbidity profile of patients attending CR are shown in Table 3**.**

**Table 3:** The comorbidity profile of patients reported by the presence and absence of depressive symptoms groups

Comorbidity	HADS < 8 Group % (*n* = 87,282)	HADS ≥ 8 Group % (*n* = 21,773)	*P*	Effect size
Angina	18.6	19.5	0.004	0.01
Arthritis	16.3	18.9	< 0.001	0.03
Cancer	7.8	7.2	0.003	0.01
Diabetes	20.5	26.7	< 0.001	0.06
Rheumatism	2.6	3.7	< 0.001	0.03
Stroke	4.5	6.6	< 0.001	0.04
Osteoporosis	2.1	2.7	< 0.001	0.02
Hypertension	49.5	49.8	0.43	0
Chronic bronchitis (COPD)	2.6	4.2	< 0.001	0.04
Emphysema	2.0	3.1	< 0.001	0.03
Asthma	8.4	10.4	< 0.001	0.03
Claudication	2.5	3.7	< 0.001	0.03
Chronic back problems	11.4	14.3	< 0.001	0.04
Anxiety	2.7	5.2	< 0.001	0.06
Family history of CVD	24.8	21.6	< 0.001	0.03
Erectile dysfunction	4.9	5.2	0.08	0.01
Hypercholesterolemia	28.8	27.9	0.006	0.01

A binominal logistic regression was performed to ascertain the impact of comorbidities and other patient characteristics on the likelihood of having new onset depressive symptoms. The logistic regression model was statistically significant, X^2^(12) = 12,429.216, *p* <  0.001. The model correctly classified 84.9% of the cases. The statistically significant variables were HADS anxiety score measurement, physical activity, weight, age, gender, marital status, IMD, diabetes, stroke, chronic back problems and total number of comorbidities. Table 4 shows the regression model.

**Table 4:** Multivariable adjusted odds ratios for new onset depressive symptoms

Variable	B	SE	Odds Ratio	Lower 95% CI for Odds Ratio	Upper 95% CI for Odds Ratio
Age	0.009	0.001	1.009	1.006	1.012
Weight	0.002	0.001	1.002	1.000	1.004
HADS anxiety score measurement	.360	0.004	1.443	1.422	1.445
150 min. a week physical activity (No)	0.626	0.031	1.870	1.761	1.985
Smoking (Yes)	0.097	0.052	1.102	0.995	1.221
Gender (Male)	0.227	0.035	1.254	1.170	1.344
Marital Status (Single)	0.138	0.034	1.148	1.074	1.227
Diabetes (Yes)	0.260	0.036	1.297	1.209	1.392
Stroke (Yes)	0.434	0.063	1.543	1.363	1.745
Chronic back problems (Yes)	0.091	0.046	1.095	1.000	1.198
Total number of comorbidities	0.029	0.011	1.029	1.008	1.051
IMD (Most deprived)	0.239	0.042	1.270	1.169	1.378

*B* regression coefficient, *SE* standard error, *CI* confidence interval for odds ratio

Increased HADS anxiety score measurement was associated with having new onset depressive symptoms at the start of CR (OR 1.443, 95% CI: 1.422, 1.445). CR participants who are physically inactive had 87% increased odds of having new onset depressive symptoms. (OR 1.870, 95% CI: 1.761, 1.985). Comorbidity of stroke, diabetes and chronic back problems were associated with presence of new onset depressive symptoms (OR 1.543, 95% CI: 1.363, 1.745; OR 1.297, 95% CI: 1.209, 1.392; OR 1.095, 95% CI: 1.000, 1.198 respectively) as well as higher total number of comorbidities (OR 1.029, 95% CI: 1.008, 1.051). In addition, CR patients from the areas of highest level of deprivation were 27% more likely to have new onset depressive symptoms at the start of CR (OR 1.270, 95% CI: 1.169, 1.378) and males were 25% more likely to have depressive symptoms (OR 1.254, 95% CI: 1.170, 1.344).

## Discussion

Previous studies have shown the association of new onset depressive symptoms with major adverse cardiac events and mortality. However, previously which factors are associated with new onset depressive symptoms had not been thoroughly examined. Therefore, the current study provides insights into the factors associated with post heart event new onset depressive symptoms. The findings of this study show the factors that are statistically significantly associated with new onset depressive symptoms are higher total number of comorbidities, increased weight, high HADS anxiety symptoms, physical inactivity and variety of comorbidities. Additionally, in terms of demographics, patients with new onset depressive symptoms were more likely to be male, single and from areas with higher social deprivation.

One finding is that patients with new onset depressive symptoms have a higher number of total comorbidities compared to patients with absence of depressive symptoms after their heart event. Likewise, higher number of comorbidities was associated with increased odds of having new onset depressive symptoms at the start of CR after accounting for other covariates (OR: 1.029, CI: 1.008, 1.051). In addition, a range of comorbidities including angina, arthritis, diabetes, rheumatism, stroke, osteoporosis, chronic bronchitis, emphysema, asthma, anxiety, claudication, and chronic back problems were found to be more prevalent in patients with new onset depressive symptoms. However, a prior RCT data driven study of Vitinius et al. [24] was not able to find an association between comorbidities and depressive symptoms. This may be due to their population being younger (mean age 59.1 ± 19.8) in comparison to ours (65.79 ± 11.14). RCTs are known to recruit a younger population, therefore recommendations have been made for RCTs to be more inclusive of older aged, multi-morbid populations [6]. Additionally, patients with multiple comorbidities are less likely to be referred to or uptake CR which creates challenges for services and health care providers [25, 26]. Increased numbers of comorbidities may limit cardiac patients’ physical functioning and thereby increase their depression levels [27]. However, patients with multiple comorbidities may benefit from attending CR which improves their functional capacity and psychosocial conditions [26, 28].

Diabetes is one of the most prevalent comorbid condition in patients with new onset depression in our sample. In addition, the current study has shown that diabetes was associated with 29% increased odds of having new onset depressive symptoms in CR attenders (OR: 1.297 95% CI: 1.209, 1.392). At the start of CR, diabetic patients presented with more cardiovascular risk factors and had reduced physical fitness [29]. The positive impact of CR in patients with diabetes has been investigated by previous studies in terms of mortality [30, 31]. Due to having a greater cardiac risk profile and lower programme uptake rate, CR programmes are recommended to target patients with diabetes [32–34]. Given that the prevalence of diabetes is continuing to rise [35], the medical management of diabetes may be relevant and lead to a reduction in depressive symptoms.

For each percent increase in the proportion of patients with stroke comorbidity, the odds of being in the new onset depressive symptoms category increases by a factor of 1.543 compared to absence of new onset depressive symptoms. The presence of stroke comorbidity is associated with a lower likelihood of a patient being referred to [25], and uptake CR [26]. Yet, stroke patients can benefit from CR programmes in terms of improvements in their cardiovascular fitness and functional capacity [36], therefore involvement of these patients into CR can be of benefit.

Our study has shown that respiratory related conditions such as chronic bronchitis, emphysema and asthma were associated with new onset depressive symptoms in baseline univariate analysis. Patients with CVD and COPD experience problems of breathlessness and disability, therefore cardiac rehabilitation services are recommended to provide sufficient flexibility to be inclusive of patients with COPD [37]. The comorbidities of arthritis, rheumatism, osteoporosis and back pain were also associated with increased depressive symptoms after heart event. The findings of a cohort study has shown that, at baseline CR assessments, patients having these comorbid conditions had a poorer health profile such as lack of physical activity and fitness than those who do not present with these comorbidities [38] which may be responsible from their increased levels of depression. Indeed, in the current study, the comorbidity of chronic back pain was associated with increased odds of having new onset depressive symptoms in patients commencing CR (OR 1.095, 95%CI: 1.000, 1.198). This study is the first to shed a light upon the variety of comorbidities and their association with new onset depressive symptoms among patients attending CR.

BMI was statistically significantly higher among patients with new onset depressive symptoms group with difference of 0.79 compared to patients with absence of new onset depressive symptoms, whereas they were in the same range of being overweight (BMI 29.00 vs 28.21). However, this statistically significant finding have not been observed in a USA based CR study [28], the reason for this might be that this American study have not factored in the patients with new onset depressive symptoms whose characteristics may be different. Patients with new onset depressive symptoms had also increased weight by 1.13 kilogrammes compared to patients with absence of new depressive symptoms group in the current study. In addition, in multivariate analysis, weight measurement remained to be significantly associated with new onset depressive symptoms after adjusting for other covariates.

The association of anxiety with depressive symptoms is confirmed by this study. The mean HADS anxiety scores were both statistically significant and clinically meaningfully higher in patients with new onset depressive symptoms group compared to absence of new onset depression (MD:5.38 (95%CI 5.33 to 5.44)). A recent study of Lemay et al. 2019 has shown that minimum clinically important difference in HADS is 1.7 for CR patients [39]. The mean difference among these groups were more than threefold higher than the minimal clinically important difference.

One clinically relevant finding was that smoking and physical inactivity were two of the modifiable cardiac risk factors that were associated with new onset depression. These results support previous systematic reviews conducted in the general population which show the prospective association between physical activity and smoking with depression [40, 41], and some cohort studies that were unable to differentiate new onset depressive symptoms in cardiac populations [42–44]. Our findings have added that at baseline CR assessments patients who present with new onset post heart event depression are more likely to smoke and be physically inactive compared to patients with absence of new onset depression. In addition in multivariable analysis physical inactivity was statistically significantly associated with 87% increased odds of having new onset depressive symptoms at baseline CR (OR 1.870, 95% CI: 1.761, 1.985). However, smoking was unable to reach the statistical significance in the regression model.

The patient demographics associated with new onset depressive symptoms were being single, which was in line with previous studies [45], male gender and being older. The English Index of Multiple Deprivation (IMD) was also one of the demographic measures included in the current study. A recent USA based study has shown that lower neighbourhood socioeconomic context, measured by neighbourhood deprivation index, was associated with reduced likelihood of CR uptake [46]. However, our study used the IMD measure and is the first to show that patients with new onset depressive symptoms attending CR were more likely to be from areas with higher levels of social deprivation. This association of increased social deprivation with new onset depressive symptoms remained significant after adjusting for other covariates by having 27% increased odds of presenting with new onset depressive symptoms (OR 1.270, 95%CI: 1.169, 1.378). Deprivation is associated with poor health behaviours such as smoking and physical inactivity [47] which might be responsible for their depressive symptoms initiation. Patients from areas with greater deprivation can be more disadvantaged naturally and experience barriers to attending CR [46] therefore strategies for inclusion of this patient group appears to be a natural next step. In addition, screening depressive symptoms in patients from areas of higher levels of deprivation that attend CR may be of benefit for early detection of the patients with high risks which perhaps could be investigated in the future studies.

Our findings are in line with the European Society of Cardiology (ESC) Position statement on psychosocial aspects of cardiac rehabilitation supporting that patients with low socioeconomic status or from areas of higher deprivation are more likely to have depressive symptoms which may lead to poor prognosis of their CVD [48]. We agree that it is important for CR programmes to identify patients with new onset depressive symptoms or other psychosocial risk factors and offer tailored interventions to these patients by a trained health care professionals [48].

### Limitations

Our population excluded patients with prior history of depression. This is because we aimed to investigate the factors associated with new onset depressive symptoms. However, when we examined the characteristics of our sample, it was representative of all available patients during the study time period ((*n* = 277,521), mean age was 65.79 compared to 65.06, 26% female compared to 27%, and this proportion did not differ by more than 4% for other variables). The study sample was nationally representative of patients with new onset depressive symptoms in the UK. The use of an observational approach enabled us to generate real world understanding by analysing routinely collected clinical data. The data included more patients with multi-comorbidities and higher female proportion than prior RCTs [6]. However, due to the nature of an observational study, causal conclusions cannot be drawn.

## Conclusion

According to the results of multivariate analysis, the characteristics that associated with new onset depressive symptoms were having higher total number of comorbidities, diabetes, stroke, chronic back problems, increased weight, physical inactivity, high anxiety scores, being male, single and from areas of greater deprivation. At baseline assessment, CR programmes need to acknowledge that patients with new onset depressive symptoms may need tailored CR intervention due to their multi-morbid medical condition and poor patient characteristics including physical inactivity, high anxiety scores, increased weight and others. Further studies need to focus on patient outcomes related to new onset depressive symptoms.

## Abbreviations

BHF: British Heart Foundation
BMI: Body mass index
CABG: Coronary artery bypass graft
CR: Cardiac rehabilitation
CVD: Cardiovascular disease
HADS: Hospital anxiety and depression scale
IMD: English index of multiple deprivation
MI: Myocardial infarction
NACR: National Audit of cardiac rehabilitation
PCI: Percutaneous coronary intervention
